# Increased risk of rhinovirus infection in children during the coronavirus disease‐19 pandemic

**DOI:** 10.1111/irv.12854

**Published:** 2021-03-14

**Authors:** Emi Takashita, Chiharu Kawakami, Tomoko Momoki, Miwako Saikusa, Kouhei Shimizu, Hiroki Ozawa, Makoto Kumazaki, Shuzo Usuku, Nobuko Tanaka, Ichiro Okubo, Hiroko Morita, Shiho Nagata, Shinji Watanabe, Hideki Hasegawa, Yoshihiro Kawaoka

**Affiliations:** ^1^ Influenza Virus Research Center National Institute of Infectious Diseases Tokyo Japan; ^2^ Yokohama City Institute of Public Health Kanagawa Japan; ^3^ Division of Virology Department of Microbiology and Immunology Institute of Medical Science University of Tokyo Tokyo Japan; ^4^ Department of Special Pathogens International Research Center for Infectious Diseases Institute of Medical Science University of Tokyo Tokyo Japan; ^5^ Department of Pathobiological Sciences School of Veterinary Medicine University of Wisconsin‐Madison Madison WI USA

**Keywords:** COVID‐19, influenza, rhinovirus, SARS‐CoV‐2, viral interference

## Abstract

**Background:**

Coronavirus disease (COVID‐19), which is caused by severe acute respiratory syndrome coronavirus 2 (SARS‐CoV‐2), was first detected in Japan in January 2020 and has spread throughout the country. Previous studies have reported that viral interference among influenza virus, rhinovirus, and other respiratory viruses can affect viral infections at the host and population level.

**Methods:**

To investigate the impact of COVID‐19 on influenza and other respiratory virus infections, we analyzed clinical specimens collected from 2244 patients in Japan with respiratory diseases between January 2018 and September 2020.

**Results:**

The frequency of influenza and other respiratory viruses (coxsackievirus A and B; echovirus; enterovirus; human coronavirus 229E, HKU1, NL63, and OC43; human metapneumovirus; human parainfluenza virus 1, 2, 3, and 4; human parechovirus; human respiratory syncytial virus; human adenovirus; human bocavirus; human parvovirus B19; herpes simplex virus type 1; and varicella‐zoster virus) was appreciably reduced among all patients during the COVID‐19 pandemic except for that of rhinovirus in children younger than 10 years, which was appreciably increased. COVID‐19 has not spread among this age group, suggesting an increased risk of rhinovirus infection in children.

**Conclusions:**

Rhinovirus infections should be continuously monitored to understand their increased risk during the COVID‐19 pandemic and viral interference with SARS‐CoV‐2.

## 1 INTRODUCTION

In Japan, coronavirus disease (COVID‐19), which is caused by severe acute respiratory syndrome coronavirus 2 (SARS‐CoV‐2), was first detected in January 2020 and has spread throughout the country. With the COVID‐19 pandemic still ongoing, the annual season of influenza and other respiratory virus epidemics has arrived. Previous studies have reported that viral interference among influenza virus, rhinovirus, and other respiratory viruses can affect viral infections at the host and population level.^1^, ^2^ To investigate the effect of COVID‐19 on these virus infections, we analyzed clinical specimens collected from patients in Japan with respiratory diseases from January 2018 through September 2020.

## 2 METHODS

### 2.1 Clinical specimens

Respiratory specimens (nasal swab, throat swab, nasal discharge, saliva, tracheal aspiration fluid, or sputum) were collected from 2244 patients with respiratory diseases in Yokohama, Japan, from January 2018 through September 2020 as part of the National Epidemiological Surveillance of Infectious Diseases and the Active Epidemiological Investigation for COVID‐19 in Japan. Of these 2244 patients, 1197 (53.3%) were men, 1044 (46.5%) were women, and 3 (0.1%) provided no sex information; 1119 (49.9%) were younger than 10 years, 1105 (49.2%) were aged 10 years or older, and 20 (0.9%) provided no age information. Specimens were negative for SARS‐CoV‐2.

### 2.2 Virus detection

Influenza virus,^3^ rhinovirus, and other respiratory viruses (coxsackievirus A and B^4^; echovirus^4^; enterovirus^4^; human coronavirus 229E, HKU1, NL63, and OC43; human metapneumovirus; human parainfluenza virus 1, 2, 3, and 4; human parechovirus^5^; human respiratory syncytial virus; human adenovirus^6^, ^7^; human bocavirus; human parvovirus B19^8^; herpes simplex virus type 1^9^; and varicella‐zoster virus^10^) were detected by using virus isolation, PCR, RT‐PCR, real‐time RT‐PCR, sequencing, FTD Respiratory pathogens 21 (Fast Track Diagnostics), or Seeplex RV15 OneStep ACE Detection (Seegene) as shown in Table 1.

**TABLE 1:** Laboratory diagnostic methods used in this study

Virus	Diagnostic method
Influenza virus	Virus isolation, rRT‐PCR
Rhinovirus	rRT‐PCR (FTD), RT‐PCR (Seeplex)
Coxsackievirus A, B	Virus isolation, RT‐PCR, Sequencing, rRT‐PCR (FTD)
Echovirus	RT‐PCR, Sequencing, rRT‐PCR (FTD)
Enterovirus	Virus isolation, RT‐PCR, Sequencing, rRT‐PCR (FTD), RT‐PCR (Seeplex)
Human coronavirus 229E, HKU1, NL63, OC43	rRT‐PCR (FTD), RT‐PCR (Seeplex)
Human metapneumovirus	rRT‐PCR (FTD), RT‐PCR (Seeplex)
Human parainfluenza virus 1, 2, 3, 4	rRT‐PCR (FTD), RT‐PCR (Seeplex)
Human parechovirus	Virus isolation, RT‐PCR, Sequencing, rRT‐PCR (FTD)
Human respiratory syncytial virus	rRT‐PCR (FTD), RT‐PCR (Seeplex)
Human adenovirus	Virus isolation, PCR, Sequencing, rRT‐PCR (FTD), RT‐PCR (Seeplex)
Human bocavirus	rRT‐PCR (FTD), RT‐PCR (Seeplex)
Human parvovirus B19	PCR, Sequencing
Herpes simplex virus type 1	Virus isolation, PCR, Sequencing
Varicella‐zoster virus	PCR, Sequencing

Abbreviations: rRT‐PCR, real‐time RT‐PCR; FTD, FTD Respiratory pathogens 21 (Fast Track Diagnostics, Sliema, Malta); Seeplex, Seeplex RV15 OneStep ACE Detection (Seegene, Seoul, Republic of Korea).

## 3 RESULTS

**FIGURE 1:**
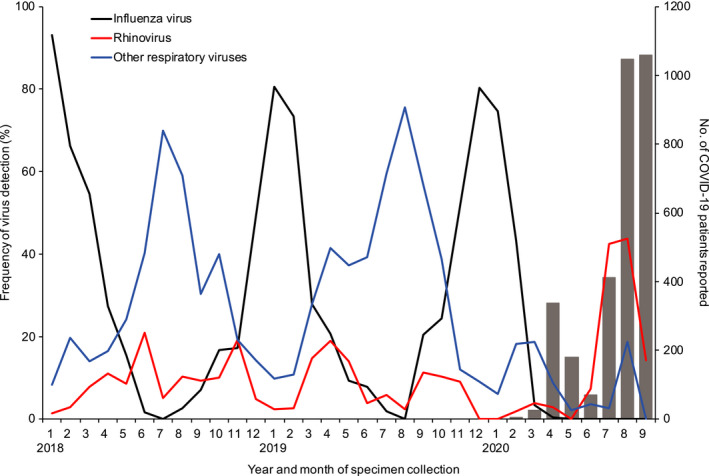
Detection of influenza virus, rhinovirus, and other respiratory viruses from January 2018 through September 2020 in Yokohama, Japan. Influenza virus (n = 592), rhinovirus (n = 155), and other respiratory viruses (coxsackievirus A and B; echovirus; enterovirus; human coronavirus 229E, HKU1, NL63, and OC43; human metapneumovirus; human parainfluenza virus 1, 2, 3, and 4; human parechovirus; human respiratory syncytial virus; human adenovirus; human bocavirus; human parvovirus B19; herpes simplex virus type 1; and varicella‐zoster virus; n = 475) were detected from 2244 patients with respiratory diseases as part of the National Epidemiological Surveillance of Infectious Diseases and the Active Epidemiological Investigation for COVID‐19 in Japan. Gray bars indicate the number of COVID‐19 patients reported by local government officials from February through September, 2020 (n = 3131)

Of the 2244 specimens, 592 influenza virus, 155 rhinovirus, and 475 other respiratory viruses were detected. Two or three viruses were co‐detected in 61 specimens. Virus‐specific seasonality was observed for influenza virus, rhinovirus, and other respiratory viruses as previously reported^2^ (Figure 1, Table S1). Influenza virus peaked in winter, whereas rhinovirus and the other respiratory viruses peaked in spring and autumn. After the first case of COVID‐19 in Yokohama was detected in February 2020, the frequency of influenza and other respiratory viruses was appreciably reduced. In contrast, the frequency of rhinovirus infection increased appreciably during the COVID‐19 pandemic. A study in Australia has also reported a high incidence of rhinovirus infection during the COVID‐19 pandemic.^11^

**FIGURE 2:**
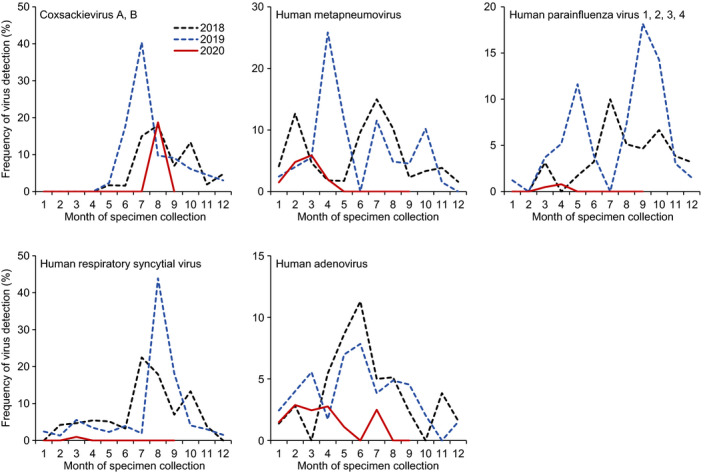
Comparison of detection of respiratory viruses from January 2018 through September 2020 in Yokohama, Japan. Coxsackievirus A and B (n = 76); human metapneumovirus (n = 105); human parainfluenza virus 1, 2, 3, and 4 (n = 56); human respiratory syncytial virus (n = 84); and human adenovirus (n = 69) were detected from 2244 patients with respiratory diseases as part of the National Epidemiological Surveillance of Infectious Diseases and the Active Epidemiological Investigation for COVID‐19 in Japan

Next, we compared the frequency of five representative respiratory viruses by year to examine whether the frequency of these viruses increased during the COVID‐19 pandemic (Figure 2). The representative viruses—coxsackievirus A and B; human metapneumovirus; human parainfluenza virus 1, 2, 3, and 4; human respiratory syncytial virus; and human adenovirus—were detected in more than 50 patients during the study period, and their maximum frequency per month was >10%. The frequency of these viruses was appreciably reduced after the COVID‐19 pandemic began. These data show that the emergence of SARS‐CoV‐2 is inversely correlated with the number of patients with other respiratory virus infections.

**FIGURE 3:**
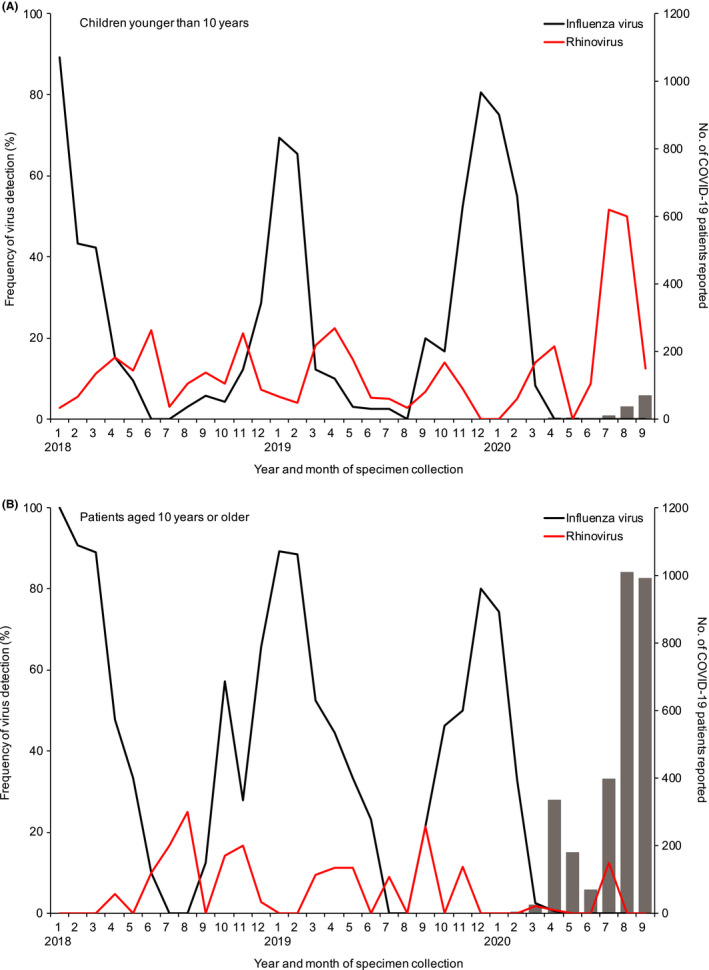
Detection of influenza virus and rhinovirus by age group from January 2018 through September 2020 in Yokohama, Japan. Influenza virus and rhinovirus were detected from children younger than 10 y (A; n = 394) and patients aged 10 y or older (B; n = 345) with respiratory diseases as part of the National Epidemiological Surveillance of Infectious Diseases and the Active Epidemiological Investigation for COVID‐19 in Japan. Gray bars indicate the number of COVID‐19 patients (A; n = 117, B; n = 3007) reported by local government officials from February 2020 through September 2020. Seven COVID‐19 patients were excluded because no age information was available

SARS‐CoV‐2 infections have been less frequent in children than in adults worldwide, including Yokohama, since the first case was detected. We compared the frequency of influenza virus and rhinovirus between children <10 years of age and patients aged 10 years or older (Figure 3, Table S1). The frequency of influenza virus was appreciably reduced in both age groups after the COVID‐19 pandemic began. However, the frequency of rhinovirus increased appreciably during the COVID‐19 pandemic in children <10 years of age but not in patients aged 10 years or older. Although COVID‐19 has not spread among children, there is an increased risk of rhinovirus infection in children.

## 4 CONCLUSIONS

To prevent SARS‐CoV‐2 infection, precautions such as physical distancing, mask wearing, keeping rooms well‐ventilated, avoiding crowds and close contact, washing hands, covering coughs and sneezes, and cleaning and disinfecting touched surfaces are recommended. These precautions also help prevent other virus infections.^12^ In this study, we found that the frequency of influenza and other respiratory viruses (coxsackievirus A and B; human metapneumovirus; human parainfluenza virus 1, 2, 3, and 4; human respiratory syncytial virus; and human adenovirus) was appreciably reduced during the COVID‐19 pandemic. These reductions might be caused by these non‐pharmaceutical interventions.

After May 2020, we did not detect enveloped viruses (ie, influenza virus; human metapneumovirus; human parainfluenza virus 1, 2, 3, and 4; and human respiratory syncytial virus), but we did detect non‐enveloped viruses, including rhinovirus; coxsackievirus A and B; and human adenovirus. This difference in detection between enveloped and non‐enveloped viruses might be related to their stability.

In this study, rhinovirus infection appreciably increased in children despite the recommended precautions. Previous studies have shown that rhinovirus can be transmitted by either fomites^13^ or aerosol.^14^ Since rhinovirus is a non‐enveloped virus, it is relatively resistant to ethanol‐containing disinfectant,^15^ and it can survive on environmental surfaces for a prolonged period of time.^13^ These viral properties might hamper the prevention of rhinovirus infection. Furthermore, Leung et al identified human coronavirus HKU1, NL63, and OC43, influenza virus, and rhinovirus in the exhaled breath and coughs of children and adults with acute respiratory illness and reported that surgical face masks could prevent the transmission of seasonal human coronaviruses and influenza viruses, but not that of rhinoviruses, from symptomatic individuals.^16^

Viral interference between influenza A virus and rhinovirus has been reported at the host and population level.^1^, ^2^ Rhinovirus infection induces an antiviral interferon response that protects against influenza A virus infection in human airway epithelial cells,^2^ supporting the potential role of virus‐induced innate immunity in driving the asynchronous circulation of rhinovirus and influenza virus. In fact, data from several European countries indicate that the rhinovirus epidemic might have interrupted and delayed the spread of the pandemic virus during the influenza A(H1N1) 2009 pandemic.^17^, ^18^, ^19^ However, the analysis of influenza and rhinovirus infections since 2018 in this study shows an inverse correlation between these two virus infections: when the number of influenza cases increases, the number of rhinovirus infections decreases, suggesting that there might be interference of rhinovirus infections by influenza virus. After SARS‐CoV‐2 emerged, we found that rhinovirus infection appreciably increased in children younger than 10 years, possibly due to the lack of influenza viruses in the population.

Our results suggest an increased risk of rhinovirus infection in children. Rhinovirus causes the common cold but can also cause severe respiratory tract infection, which may be followed by pulmonary and extrapulmonary complications in some patients.^20^ Previous studies reported that rhinovirus is more likely to cause severe disease in winter, although peak prevalence occurs in the spring and autumn.^21^, ^22^ Consequently, rhinovirus infections should be continuously monitored to understand their increased risk during the COVID‐19 pandemic and viral interference with SARS‐CoV‐2.

## CONFLICT OF INTEREST

None declared.

## AUTHOR CONTRIBUTIONS

**Emi Takashita:** Conceptualization (equal); Data curation (equal); Formal analysis (equal); Investigation (equal); Methodology (equal); Writing‐original draft (equal). **Chiharu Kawakami:** Conceptualization (equal); Data curation (equal); Formal analysis (equal); Investigation (equal); Methodology (equal); Writing‐original draft (equal). **Tomoko Momoki:** Formal analysis (equal); Investigation (equal); Writing‐review & editing (supporting). **Miwako Saikusa:** Formal analysis (equal); Investigation (equal); Writing‐review & editing (supporting). **Kouhei Shimizu:** Formal analysis (equal); Investigation (equal); Writing‐review & editing (supporting). **Hiroki Ozawa:** Formal analysis (equal); Investigation (equal); Writing‐review & editing (supporting). **Makoto Kumazaki:** Formal analysis (equal); Investigation (equal); Writing‐review & editing (supporting). **Shuzo Usuku:** Formal analysis (equal); Investigation (equal); Writing‐review & editing (supporting). **Nobuko Tanaka:** Writing‐review & editing (supporting). **Ichiro Okubo:** Writing‐review & editing (supporting). **Hiroko Morita:** Formal analysis (equal); Investigation (equal); Writing‐review & editing (supporting). **Shiho Nagata:** Formal analysis (equal); Investigation (equal); Writing‐review & editing (supporting). **Shinji Watanabe:** Writing‐review & editing (supporting). **Hideki Hasegawa:** Funding acquisition (lead); Writing‐review & editing (supporting). **Yoshihiro Kawaoka:** Conceptualization (lead); Data curation (lead); Funding acquisition (lead); Methodology (lead); Project administration (lead); Writing‐review & editing (lead).

## Supporting information

Table S1

## Data Availability

The data that supports the findings of this study are available in the supplementary material of this article.

## References

[1] Nickbakhsh S , Mair C , Matthews L , et al. Virus‐virus interactions impact the population dynamics of influenza and the common cold. Proc Natl Acad Sci USA. 2019;116(52):27142‐27150. 10.1073/pnas.1911083116 PMC6936719 31843887

[2] Wu A , Mihaylova VT , Landry ML , Foxman EF . Interference between rhinovirus and influenza A virus: a clinical data analysis and experimental infection study. Lancet Microbe. 2020;1(6):e254‐e262. 33103132 10.1016/s2666-5247(20)30114-2 PMC7580833

[3] World Health Organization . Manual for the Laboratory Diagnosis and Virological Surveillance of Influenza. https://www.who.int/influenza/gisrs_laboratory/manual_diagnosis_surveillance_influenza/en/. Accessed February 12, 2021.

[4] Momoki TS . Surveillance of enterovirus infections in Yokohama city from 2004 to 2008. Jpn J Infect Dis. 2009;62(6):471‐473. 19934543

[5] Momoki TS . Analysis of human parechovirus genotypes in Yokohama district from 2000 to 2016. Jpn J Infect Dis. 2018;71(4):298‐301. 29709970 10.7883/yoken.JJID.2017.490

[6] Takeuchi S , Itoh N , Uchio E , Aoki K , Ohno S . Serotyping of adenoviruses on conjunctival scrapings by PCR and sequence analysis. J Clin Microbiol. 1999;37(6):1839‐1845. 10325334 10.1128/jcm.37.6.1839-1845.1999 PMC84965

[7] Fujimoto T , Matsushima Y , Shimizu H , et al. A molecular epidemiologic study of human adenovirus type 8 isolates causing epidemic keratoconjunctivitis in Kawasaki City, Japan in 2011. Jpn J Infect Dis. 2012;65(3):260‐263. 22627311 10.7883/yoken.65.260

[8] Mosquera Mdel M , de Ory F , Moreno M , Echevarria JE . Simultaneous detection of measles virus, rubella virus, and parvovirus B19 by using multiplex PCR. J Clin Microbiol. 2002;40(1):111‐116. 11773102 10.1128/JCM.40.1.111-116.2002 PMC120129

[9] Tyler KL . Herpes simplex virus infections of the central nervous system: encephalitis and meningitis, including Mollaret's. Herpes. 2004;11(Suppl 2):57A‐64A. 15319091

[10] Loparev VN , Argaw T , Krause PR , Takayama M , Schmid DS . Improved identification and differentiation of varicella‐zoster virus (VZV) wild‐type strains and an attenuated varicella vaccine strain using a VZV open reading frame 62‐based PCR. J Clin Microbiol. 2000;38(9):3156‐3160. 10970349 10.1128/jcm.38.9.3156-3160.2000 PMC87343

[11] Sullivan SG , Carlson S , Cheng AC , et al. Where has all the influenza gone? The impact of COVID‐19 on the circulation of influenza and other respiratory viruses, Australia, March to September 2020. Euro Surveill. 2020;25(47):2001847. 10.2807/1560-7917.ES.2020.25.47.2001847 PMC7693168 33243355

[12] Sherman AC , Babiker A , Sieben AJ , et al. The effect of SARS‐CoV‐2 mitigation strategies on seasonal respiratory viruses: a tale of two large metropolitan centers in the United States. Clin Infect Dis. 2021;72(5):e154–e157. 33161424 10.1093/cid/ciaa1704 PMC7717225

[13] Winther B , McCue K , Ashe K , Rubino JR , Hendley JO . Environmental contamination with rhinovirus and transfer to fingers of healthy individuals by daily life activity. J Med Virol. 2007;79(10):1606‐1610. 17705174 10.1002/jmv.20956

[14] Dick EC , Jennings LC , Mink KA , Wartgow CD , Inhorn SL . Aerosol transmission of rhinovirus colds. J Infect Dis. 1987;156(3):442‐448. 3039011 10.1093/infdis/156.3.442

[15] Savolainen‐Kopra C , Korpela T , Simonen‐Tikka ML , et al. Single treatment with ethanol hand rub is ineffective against human rhinovirus–hand washing with soap and water removes the virus efficiently. J Med Virol. 2012;84(3):543‐547. 22246844 10.1002/jmv.23222

[16] Leung NHL , Chu DKW , Shiu EYC , et al. Respiratory virus shedding in exhaled breath and efficacy of face masks. Nat Med. 2020;26(5):676‐680. 32371934 10.1038/s41591-020-0843-2 PMC8238571

[17] Linde A , Rotzen‐Ostlund M , Zweygberg‐Wirgart B , Rubinova S , Brytting M . Does viral interference affect spread of influenza? Euro Surveill. 2009;14(40):19354. 19822124

[18] Casalegno JS , Ottmann M , Duchamp MB , et al. Rhinoviruses delayed the circulation of the pandemic influenza A (H1N1) 2009 virus in France. Clin Microbiol Infect. 2010;16(4):326‐329. 20121829 10.1111/j.1469-0691.2010.03167.x

[19] Anestad G , Nordbo SA . Virus interference. Did rhinoviruses activity hamper the progress of the 2009 influenza A (H1N1) pandemic in Norway? Med Hypotheses. 2011;77(6):1132‐1134. 21975051 10.1016/j.mehy.2011.09.021

[20] To KKW , Yip CCY , Yuen KY . Rhinovirus ‐ from bench to bedside. J Formos Med Assoc. 2017;116(7):496‐504. 28495415 10.1016/j.jfma.2017.04.009

[21] Lee WM , Lemanske RF Jr , Evans MD , et al. Human rhinovirus species and season of infection determine illness severity. Am J Respir Crit Care Med. 2012;186(9):886‐891. 22923659 10.1164/rccm.201202-0330OC PMC3530215

[22] To KK , Lau SK , Chan KH , et al. Pulmonary and extrapulmonary complications of human rhinovirus infection in critically ill patients. J Clin Virol. 2016;77:85‐91. 26921740 10.1016/j.jcv.2016.02.014

