# Supplementary material for: Increased risk of rhinovirus infection in children during the coronavirus disease‐19 pandemic
Source: Influenza Other Respir Viruses. 2021 Mar 14;15(4):488–94. doi: 10.1111/irv.12854 (PMC8189209; doi:10.1111/irv.12854)
Supplement: Supplementary file 1 — Table S1 [file IRV-15-488-s001.docx]

**Table S1.** Number of influenza virus, rhinovirus, and other respiratory viruses detected from January 2018 through September 2020 in Yokohama, Japan

| Age group | | 2018 | | | | | | | | | | | | 2019 | | | | | | | | | | | | 2020 | | | | | | | | |
| --- | --- | --- | --- | --- | --- | --- | --- | --- | --- | --- | --- | --- | --- | --- | --- | --- | --- | --- | --- | --- | --- | --- | --- | --- | --- | --- | --- | --- | --- | --- | --- | --- | --- | --- |
|  |  | Jan | Feb | Mar | Apr | May | Jun | Jul | Aug | Sep | Oct | Nov | Dec | Jan | Feb | Mar | Apr | May | Jun | Jul | Aug | Sep | Oct | Nov | Dec | Jan | Feb | Mar | Apr | May | Jun | Jul | Aug | Sep |
| All ages | Specimens tested | 73 | 71 | 64 | 55 | 58 | 62 | 40 | 39 | 43 | 30 | 52 | 63 | 82 | 75 | 54 | 58 | 43 | 51 | 52 | 41 | 44 | 49 | 66 | 66 | 67 | 104 | 204 | 253 | 181 | 27 | 40 | 16 | 21 |
|  | Influenza virus | 68 | 47 | 35 | 15 | 9 | 1 | 0 | 1 | 3 | 5 | 9 | 31 | 66 | 55 | 15 | 12 | 4 | 4 | 1 | 0 | 9 | 12 | 34 | 53 | 50 | 45 | 7 | 1 | 0 | 0 | 0 | 0 | 0 |
|  | Rhinovirus | 1 | 2 | 5 | 6 | 5 | 13 | 2 | 4 | 4 | 3 | 10 | 3 | 2 | 2 | 8 | 11 | 6 | 2 | 3 | 1 | 5 | 5 | 6 | 0 | 0 | 2 | 8 | 7 | 0 | 2 | 17 | 7 | 3 |
|  | Other respiratory viruses | 6 | 14 | 9 | 9 | 14 | 25 | 28 | 23 | 13 | 12 | 10 | 9 | 8 | 8 | 15 | 24 | 16 | 20 | 31 | 31 | 25 | 19 | 8 | 6 | 4 | 19 | 38 | 22 | 4 | 1 | 1 | 3 | 0 |
| <10 years | Specimens tested | 37 | 37 | 45 | 33 | 42 | 50 | 32 | 34 | 35 | 23 | 33 | 28 | 36 | 49 | 33 | 40 | 34 | 38 | 41 | 36 | 30 | 36 | 40 | 31 | 32 | 40 | 36 | 28 | 26 | 23 | 31 | 14 | 16 |
|  | Influenza virus | 33 | 16 | 19 | 5 | 4 | 0 | 0 | 1 | 2 | 1 | 4 | 8 | 25 | 32 | 4 | 4 | 1 | 1 | 1 | 0 | 6 | 6 | 21 | 25 | 24 | 22 | 3 | 0 | 0 | 0 | 0 | 0 | 0 |
|  | Rhinovirus | 1 | 2 | 5 | 5 | 5 | 11 | 1 | 3 | 4 | 2 | 7 | 2 | 2 | 2 | 6 | 9 | 5 | 2 | 2 | 1 | 2 | 5 | 3 | 0 | 0 | 2 | 5 | 5 | 0 | 2 | 16 | 7 | 2 |
|  | Other respiratory viruses | 5 | 13 | 9 | 7 | 12 | 23 | 24 | 23 | 13 | 12 | 9 | 8 | 7 | 8 | 12 | 23 | 12 | 17 | 29 | 30 | 22 | 17 | 8 | 4 | 2 | 9 | 13 | 10 | 2 | 1 | 1 | 2 | 0 |
| ≥10 years | Specimens tested | 34 | 32 | 18 | 21 | 15 | 10 | 6 | 4 | 8 | 7 | 18 | 35 | 46 | 26 | 21 | 18 | 9 | 13 | 11 | 5 | 14 | 13 | 26 | 35 | 35 | 61 | 167 | 225 | 155 | 4 | 8 | 2 | 3 |
|  | Influenza virus | 34 | 29 | 16 | 10 | 5 | 1 | 0 | 0 | 1 | 4 | 5 | 23 | 41 | 23 | 11 | 8 | 3 | 3 | 0 | 0 | 3 | 6 | 13 | 28 | 26 | 20 | 4 | 1 | 0 | 0 | 0 | 0 | 0 |
|  | Rhinovirus | 0 | 0 | 0 | 1 | 0 | 1 | 1 | 1 | 0 | 1 | 3 | 1 | 0 | 0 | 2 | 2 | 1 | 0 | 1 | 0 | 3 | 0 | 3 | 0 | 0 | 0 | 3 | 2 | 0 | 0 | 1 | 0 | 0 |
|  | Other respiratory viruses | 1 | 1 | 0 | 1 | 1 | 1 | 4 | 0 | 0 | 0 | 0 | 1 | 1 | 0 | 3 | 1 | 4 | 3 | 2 | 1 | 3 | 2 | 0 | 2 | 2 | 10 | 25 | 12 | 2 | 0 | 0 | 1 | 0 |
